# Supplementary material for: Overexpression of the Apple (Malus × domestica) MdERF100 in Arabidopsis Increases Resistance to Powdery Mildew
Source: Int J Mol Sci. 2021 May 27;22(11):5713. doi: 10.3390/ijms22115713 (PMC8197995; doi:10.3390/ijms22115713)
Supplement: Supplementary file 1 [file ijms-22-05713-s001.zip › ijms-1211893-supplementary.pdf]

Supplementary Materials

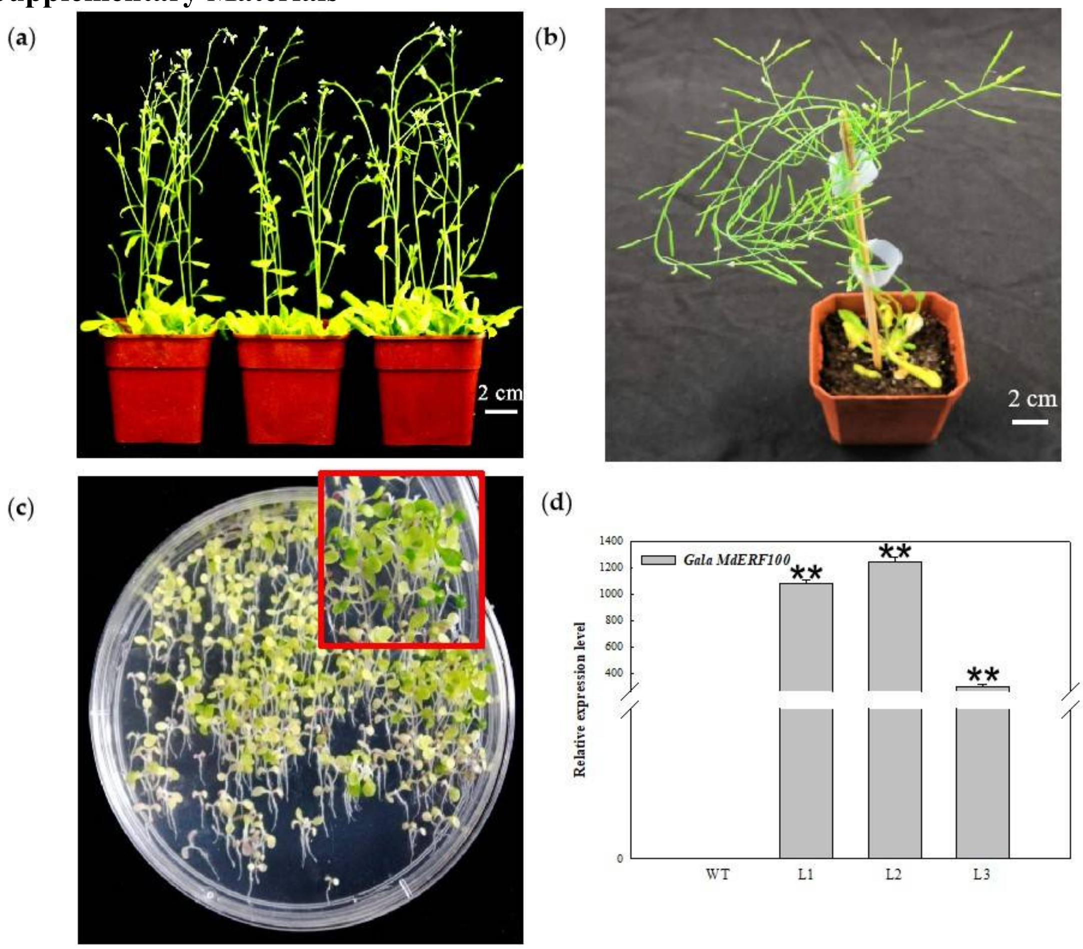

Figure S1: The selection of *Arabidopsis* lines overexpressing *MdERF100* and relative transcription level of *MdERF100*

Supplementary Table S1. qRT-PCR system

Ingredient	Volume (μL)
2×ChamQ SYBR qPCR Master Mix	10.0
Primer1	0.4
Primer2	0.4
50×ROX reference Dye 1	0.4
cDNA	1.0
ddH_2O	7.8
Total	20.0

Supplementary Table S2. Related primers sequence designed in this experiment.

Name	Forward (F) and Reverse (R) primer sequence (5'-3')	Purpose
GC-*MdERF100*-F	ATGTACGGACAGAGTGGAGCTGACT	Gene clone
GC-*MdERF100*-R	TCAGACCACCAATAACTGCTCGCCA	

GC-*MdbHLH92*-F	ATGGACCAATATATCCTCAAAGCAT	
GC-*MdbHLH92*-R	TCACCTTCCTGGAAATGGAAAACGC	
qRT-*MdERF100*-F	TGTTCCTACGGCTTTTCGGT	qRT-PCR
qRT-*MdERF100*-R	TGTAATGCTTCCCCTTCGCT	
qRT-*MdbHLH92*-F	AAATGAAGCCGAACCAGAC	
qRT-*MdbHLH92*-R	TGATGTAGGATTAGCCACC	
qRT-*MdTubulin*-F	AGGATGCTACAGCCGATGAG	
qRT-*MdTubulin*-R	GCCGAAGAACTGACGAGAATC	
qRT-*AtNPR1*-F	GCTCTGCTCGTCAATGGTTATC	
qRT-*AtNPR1*-R	GAGGAGTCGGTGTTATCGGTA	
qRT-*AtPR2*-F	CGGTACATCAACGTTGGAA	
qRT-*AtPR2*-R	GCGTAGTCTAGATGGATGTT	
qRT-*AtPR3*-F	CGCTTGTCCTGCTAGAGGTT	
qRT-*AtPR3*-R	GCTCGGTTCACAGTAGTCTGA	
qRT-*AtPDF1.2*-F	TCATGGCTAAGTTTGCTTCC	
qRT-*AtPDF1.2*-R	AATACACACGATTTAGCACC	
qRT-*AtEDS1*-F	TCATACGCAATCCAAATGTTTAC	
qRT-*AtEDS1*-R	AAAAACCTCTCTTGCTCGATCAC	
qRT-*AtPR5*-F	GCACAGAGACACACACAAAA	
qRT-*AtPR5*-R	TGTTCCTTAGAGTGAAGTCTG	

Name	Forward (F) and Reverse (R) primer sequence (5'-3')	Purpose
qRT-*AtActin1*-F	TCAATCCAGGAGATGTTTAGG	
qRT-*AtActin1*-R	ACTGCTGGTACTCTGCGACA	
MdERF100-GFP-F-*Kpn*I	acgggggacgagctcggtaccATGTACGGACAGAGTGGAGCTGACT	pCAMBIA2300-GFP
MdERF100-GFP-R-*Bam*HI	ggtgtcgactctagaggatccTCAGACCACCAATAACTGCTCGCCA	
MdbHLH92-GFP-F-*Kpn*I	acgggggacgagctcggtaccATGGACCAATATATCCTCAAAGCAT	
MdbHLH92-GFP-R-*Bam*HI	ggtgtcgactctagaggatccTCACCTTCCTGGAAATGGAAAACGC	
MdERF100-BD-F-*Eco*RI	atggccatggaggccgaattcATGTACGGACAGAGTGGAGCTGACT	pGBKT7
MdERF100-BD-R-*Bam*HI	ccgctgcaggtcgacggatccTCAGACCACCAATAACTGCTCGCCA	
MdbHLH92-BD-F-*Eco*RI	atggccatggaggccgaattcATGGACCAATATATCCTCAAAGCCT	
MdbHLH92-BD-R-*Bam*HI	ccgctgcaggtcgacggatccCTACCTTCCTGGAAATGGAAAACGC	
MdCSC1-AD-F-*Eco*RI	gccatggaggccagtgaattcATGGCGACTCTGGGTGACATTGGGG	pGADT7
MdCSC1-AD-R-*Bam*HI	cagctcgagctcgatggatccTCAATGATCTTCGTTGTGGTAGGCA	
MdBIP-AD-F-*Eco*RI	gccatggaggccagtgaattcATGGCTGGCTCTTGGAGAGCTCGCG	
MdBIP-AD-R-*Bam*HI	cagctcgagctcgatggatccTTAAAGCTCGTCATGCGACTCATCG	

MdbHLH92-AD-F-*Eco*RI	gccatggaggccagt<u>gaattc</u>ATGGACCAATATATCCTCAAAGCAT	
MdbHLH92-AD-R-*Bam*HI	cagctcgagctcgat<u>ggatcc</u>TCACCTTCCTGGAAATGGAAAACGC	
MdPAE1-AD-F-*Eco*RI	gccatggaggccagt<u>gaattc</u>ATGTTTCTCACAAGGACTGAGTATG	
MdPAE1-AD-R-*Bam*HI	cagctcgagctcgat<u>ggatcc</u>TTATAGGCGAGCAATGACGGCCTCC	
MdUBQ-AD-F-*Eco*RI	gccatggaggccagt<u>gaattc</u>ATGGCCATGTATATCCGTGTTAAGC	
MdUBQ-AD-R-*Bam*HI	cagctcgagctcgat<u>ggatcc</u>TCACCAATTGCCTGCATCTGCATCA	
MdbHLH92-NE-F-*Xho*I	agtggatccgtcgac<u>ctcgag</u>ATGGACCAATATATCCTCAAAGCAT	pUC-NE
MdbHLH92-NE-R-*Kpn*I	ctcctacccgggagc<u>ggtacc</u>TCACCTTCCTGGAAATGGAAAACGC	
MdERF100-NE-F-*Xho*I	agtggatccgtcgac<u>ctcgag</u>ATGTACGGACAGAGTGGAGCTGACT	
MdERF100-NE-R-*Kpn*I	ctcctacccgggagc<u>ggtacc</u>TCAGACCACCAATAACTGCTCGCCA	
MdERF100-CE-F-*Xho*I	gggactctagaggat<u>ctcgag</u>ATGTACGGACAGAGTGGAGCTGACT	pUC-CE
MdERF100-CE-R-*Kpn*I	atcgtatgggtacat<u>ggtacc</u>GACCACCAATAACTGCTCGCCACGT	
MdbHLH92-CE-F-*Xho*I	agtggatccgtcgac<u>ctcgag</u>ATGGACCAATATATCCTCAAAGCCT	
MdbHLH92-CE-R-*Kpn*I	ctcctacccgggagc<u>ggtacc</u>CCTTCCTGGAAATGGAAAACGCAGT	
